# Skin autofluorescence predicts major adverse cardiovascular events in patients with type 1 diabetes: a 7-year follow-up study

**DOI:** 10.1186/s12933-018-0718-8

**Published:** 2018-06-08

**Authors:** C. Blanc-Bisson, F. L. Velayoudom-Cephise, A. Cougnard-Gregoire, C. Helmer, K. Rajaobelina, C. Delcourt, L. Alexandre, L. Blanco, K. Mohammedi, M. Monlun, V. Rigalleau

**Affiliations:** 1 Nutrition Diabetology Unit, CHU Bordeaux, Haut-Levêque Hospital, Avenue Magellan, 33600 Pessac Cedex, France; 2 INSERM, Bordeaux Population Health Research Center, Team LEHA, UMR 1219, Univ Bordeaux, 33000 Bordeaux, France

**Keywords:** Skin autofluorescence, Macroangiopathy, Advanced glycation end-products, Type 1 diabetes

## Abstract

**Background:**

Advanced glycation end-products play a role in diabetic vascular complications. Their optical properties allow to estimate their accumulation in tissues by measuring the skin autofluorescence (SAF). We searched for an association between SAF and major adverse cardiovascular events (MACE) incidence in subjects with Type 1 Diabetes (T1D) during a 7 year follow-up.

**Methods:**

During year 2009, 232 subjects with T1D were included. SAF measurement, clinical [age, sex, body mass index (BMI), comorbidities] and biological data (HbA1C, blood lipids, renal parameters) were recorded. MACE (myocardial infarction, stroke, lower extremity amputation or a revascularization procedure) were registered at visits in the center or by phone call to general practitioners until 2016.

**Results:**

The participants were mainly men (59.5%), 51.5 ± 16.7 years old, with BMI 25.0 ± 4.1 kg/m^2^, diabetes duration 21.5 ± 13.6 years, HbA1C 7.6 ± 1.1%. LDL cholesterol was 1.04 ± 0.29 g/L, estimated Glomerular Filtration Rates (CKD-EPI): 86.3 ± 26.6 ml/min/1.73 m^2^. Among these subjects, 25.1% were smokers, 45.3% had arterial hypertension, 15.9% had elevated AER (≥ 30 mg/24 h), and 9.9% subjects had a history of previous MACE. From 2009 to 2016, 22 patients had at least one new MACE: 6 myocardial infarctions, 1 lower limb amputation, 15 revascularization procedures. Their SAF was 2.63 ± 0.73 arbitrary units (AU) vs 2.08 ± 0.54 for other patients (p = 0.002). Using Cox-model, after adjustment for age (as the scale time), sex, diabetes duration, BMI, hypertension, smoking status, albumin excretion rates, statin treatment and a previous history of MACE, higher baseline levels of SAF were significantly associated with an increased risk of MACE during follow-up (HR = 4.13 [1.30–13.07]; p = 0.02 for 1 AU of SAF) and Kaplan–Meier curve follow-up showed significantly more frequent MACE in group with SAF upper the median (p = 0.001).

**Conclusion:**

A high SAF predicts MACE in patients with T1D.

**Electronic supplementary material:**

The online version of this article (10.1186/s12933-018-0718-8) contains supplementary material, which is available to authorized users.

## Introduction

Despite the decline of cardiovascular disease in patients with type 1 diabetes (T1D) [1], it remains by far the first cause of mortality, as recently reported in patients with long duration of diabetes [2], and a major concern even in children who often present features of subclinical cardiovascular disease [3]. The cardiovascular risk of subjects with T1D is influenced by modifiable conventional risk factors such as arterial hypertension, dyslipidemia, and smoking, [4], and is strongly higher in the presence of diabetic nephropathy [5], but hyperglycaemia by itself is thought to play an important role.

Long-term hyperglycaemia promotes cardiovascular mortality, with a gradual increase of hazard ratios for death among patients with T1D compared to non diabetic subjects according to the HbA1C levels [6]. However a risk persists for well-controlled subjects with HbA1C ≤ 6.9%. The effect of intensive glucose control on cardiovascular events was significant only 11 years after the intensive glucose control intervention in the Diabetes Control and Complications Trial, owing to a “glucose memory” effect [7]. A recent detailed analysis of cardiovascular risk factors of the DCCT-EDIC participants emphasized the importance of the mean time-weighted HbA1C [8].

Advanced glycation end-products (AGEs) generated from excess glucose have deleterious effects on endothelial cells [9]. They are thought to contribute to the memory of hyperglycaemia [10]. The skin concentrations of AGEs have been related to the progression of intima-media thickness in the DCCT-EDIC study [11]. Due to their fluorescent properties, the accumulation of AGEs in tissues can be simply estimated by measuring the skin autofluorescence (SAF) [12]. SAF predicted later cardiovascular events in patients with type 2 diabetes [13]. We have related it to later impaired renal function [14] and neuropathy [15] in patients with T1D. However, the relation between SAF and later cardiovascular events has not been reported yet in T1D. We aimed to test the association between SAF and future occurrence of major adverse cardiovascular event (MACE) in patients with T1D followed-up over 7 years.

## Subjects and methods

### Design and patients

Two hundred and thirty-two patients with T1D were included consecutively in 2009 after consent during annual visit in University Hospital of Bordeaux. The only exclusion criterion was ultraviolet reflectance < 10% that precluded the measurement of SAF. The patients with skin phototypes V and VI were therefore not included. The following data were recorded at inclusion on the day of SAF measurement: age, sex, body mass index, duration of diabetes, treatment by Continuous Subcutaneous Insulin Infusion, arterial hypertension (blood pressure ≥ 140/90 mmHg and/or treatment with antihypertensive drug), treatment by statins, previous macrovascular events, and smoking history. The biological data recorded included HbA1c levels, blood lipids, the Albumin Excretion Rates (AER), and serum creatinine to estimate the Glomerular Filtration Rates calculated with the CKD-EPI formula [16]. Participants were prospectively followed-up until the occurrence of new cases of major adverse cardiovascular events (MACE) till March 2017. Number of patients was mentioned when data were missing.

### Skin autofluorescence (SAF)

The accumulation of AGEs was evaluated at inclusion [12], using the AGE-Reader (diagnOptics BV, Groningen, The Netherlands). The device illuminated 1 cm^2^ of the forearm skin. SAF values were calculated by dividing the mean emitted light intensity (excitation light source ranging from 300 to 420 nm) by the mean reflected excitation light intensity from the skin (over 300–420 nm). The patients with Fitzpatrick phototypes V and VI were not included due to their skin pigmentation, which had ultraviolet reflectance of < 10%. The results were the mean of three consecutive measurements of SAF and were expressed in arbitrary units (AU).

### Outcomes

MACE were defined as myocardial infarction, stroke, lower limb amputation, or a revascularization procedure for coronary, carotid or lower limb arteries. Every year, patients came in visit at hospital, MACE was collected by physician if occurred since previous visit and recorded in the informatic medical folder of each patient. If we had no recent news we called practitioner to ask if MACE or death occurred.

### Statistical methods

The results are presented as mean ± SD for continuous variables, or median (IQR) for those with skewed distribution. Categorial variables are presented as numbers (percentages). A Cox model with delayed entry (taking age as the scale-time) was used to investigate the association between SAF and MACE. R2 of the model has been calculated by the following formula: R2 = 1 − e ^− (LRT/n)^, where LRT is the likelihood-ratio statistic. Log-linearity of the model has been tested using SAF as a continuous variable and in quartile. Risk proportionality has been verified both by testing the interaction between SAF and time and by using Schoenfeld residuals. In a first step, analyses were performed to test the association between each co-variable and MACE in order to select variables for the multivariate analysis. Then, all the variables associated with MACE with a p value ≤ 0.10 were retained in the multivariate Cox model. All statistical analyses were performed using SAS version 9.3 (SAS Institute Inc, Cary, NC) and results were considered significant when p < 0.05. Follow-up was analysed using Kaplan–Meier curve.

## Results

### Characteristics of the population at inclusion

Participants were mainly men (59.5%), 51.5 ± 16.7 years old, with a 21.5 ± 13.6 years diabetes duration. Their BMI was 25.0 ± 4.1 kg/m^2^, HbA1c 7.6 ± 1.1%, eGFR was 86.0 ± 26.3 ml/min/1.73 m^2^. Among them, 25.1% were smokers, 45.3% had arterial hypertension, 15.9% had elevated AER (≥ 30 mg/24H), and 9.9% had a history of previous macrovascular event. Their SAF was 2.13 ± 0.58 AU. The scatterplot for SAF over age is presented as a Additional file 1: Figure S1.

### Subjects with new major adverse cardiovascular events during the follow-up

Twenty-two patients presented MACE during a median (IQR) duration of follow-up of 7.8 [7.6–8.0] years: 6 myocardial infarctions, 1 gangrene and 15 revascularization procedures. As compared to subjects without MACE, they were significantly more men, 10 years older, with 9 more years of diabetes duration, but similar HbA1c (Table 1). The lipid profiles did not significantly differ, but subjects with MACE were more likely to be treated by statins in comparison with subjects without MACE. Arterial hypertension, abnormal eGFR (< 60 ml/min/1.73 m^2^) and abnormal (> 30 mg/24 h) AER were significantly more frequent.

**Table 1:** Characteristics of participants at baseline according to the incidence of major adverse cardiovascular event over the 7-year follow-up

	Major cardiovascular event		
	No (N = 210)	Yes (N = 22)	p
Age (years)	50.54 ± 16.76	60.56 ± 13.30	0.007
Sex men	121 (57.6)	17 (77.2)	0.07
BMI (kg/m^2^)	24.7 ± 3.7	27.1 ± 6.3	0.09
Diabetes duration (years)	20.6 ± 12.8	29.6 ± 17.8	0.03
HbA1c (mmol/ml) in 2009 (%)	7.6 ± 1.0	7.5 ± 1.0	0.74
Insulin pump	40 (19.0)	5 (22.7)	0.77
Smoking habits (n = 219)	51 (25.8)	4 (18.1)	0.43
HDL (g/l) (n = 222)	0.64 ± 0.19	0.58 ± 0.17	0.17
LDL (g/l) (n =t221)	1.03 ± 0.29	1.04 ± 0.33	0.88
Triglycerides (g/l) (n = 205)	0.89 ± 0.63	1.11 ± 0.53	0.13
Statin at baseline (n = 222)	45 (22.5)	13 (59.0)	0.0002
Hypertension	87 (41.43)	18 (81.82)	0.0003
Previous history of MACE	13 (6.19)	10 (45.45)	< 0.0001
eGFR (mL/min) (n = 225)			
≥ 60	179 (88.18)	13 (59.09)	0.001
< 60	24 (11.82)	9 (40.91)	
Albumin excretion rate (mg/24 h) (n = 227)			
< 30	179 (86.89)	12 (57.14)	0.002
≥ 30	27 (13.11)	9 (42.86)	
SAF (AU)	2.08 (0.54)	2.63 (0.73)	0.002

Data presented in N (%) and mean ± standard deviation

*p* value: COX test for qualitative variables and quantitative variables

### SAF and new major adverse cardiovascular events (Table 2, Fig. 1)

At inclusion, the SAF was 2.63 ± 0.73 AU in patients who presented MACE during the follow-up, *vs* 2.08 ± 0.54 for the others (p = 0.002). In addition to SAF, gender, duration of diabetes, BMI, hypertension, use of statins, smoking, renal parameters, and previous MACE were associated with MACE during the follow-up at a p value ≤ 0.10 and were retained in the multivariate analysis. SAF high level in 2009 was associated with significant increased risk of MACE during the 7 next years: HR = 4.13 [1.30;13.07] for increasing of +1 SAF AU (p = 0.02). Elevated hazard ratio for sex, MACE at baseline, BMI, statins treatment, abnormal eGFR, albumin excretion rates, hypertension and diabetes duration were not significantly associated with MACE. Using Kaplan–Meier curve, the follow-up of patients categorized in two groups according to SAF median (< 2.0 vs ≥ 2.0) showed that patients with high SAF more frequently developed MACE than those with SAF lower than median (p = 0.001). R2 of the model was estimated at 19%.

**Table 2:** Risk of major adverse cardiovascular event over the follow-up according to SAF at baseline

	HR	95% Confidence interval	p
SAF (per 1 AU)	4.13	1.30; 13.07	0.02
Sex			
Men	4.15	0.87; 19.77	0.07
BMI (per 1 kg/m^2^)	1.11	0.96; 1.27	0.16
MACE at baseline			
Yes	3.01	0.99; 9.08	0.05
Statins baseline			
Yes	2.55	0.77; 8.47	0.13
eGFR (mL/min)			
< 60	1.54	0.32; 7.31	0.59
Albumin excretion rate (mg/24 h)			
≥ 30	1.92	0.54; 6.88	0.32
Hypertension			
Yes	2.70	0.61; 11.98	0.19
Diabetes duration in 2009 (per 1 year)	1.02	0.98; 1.06	0.38

Cox multivariate analysis (n = 205, 21 events)

**Fig. 1:**
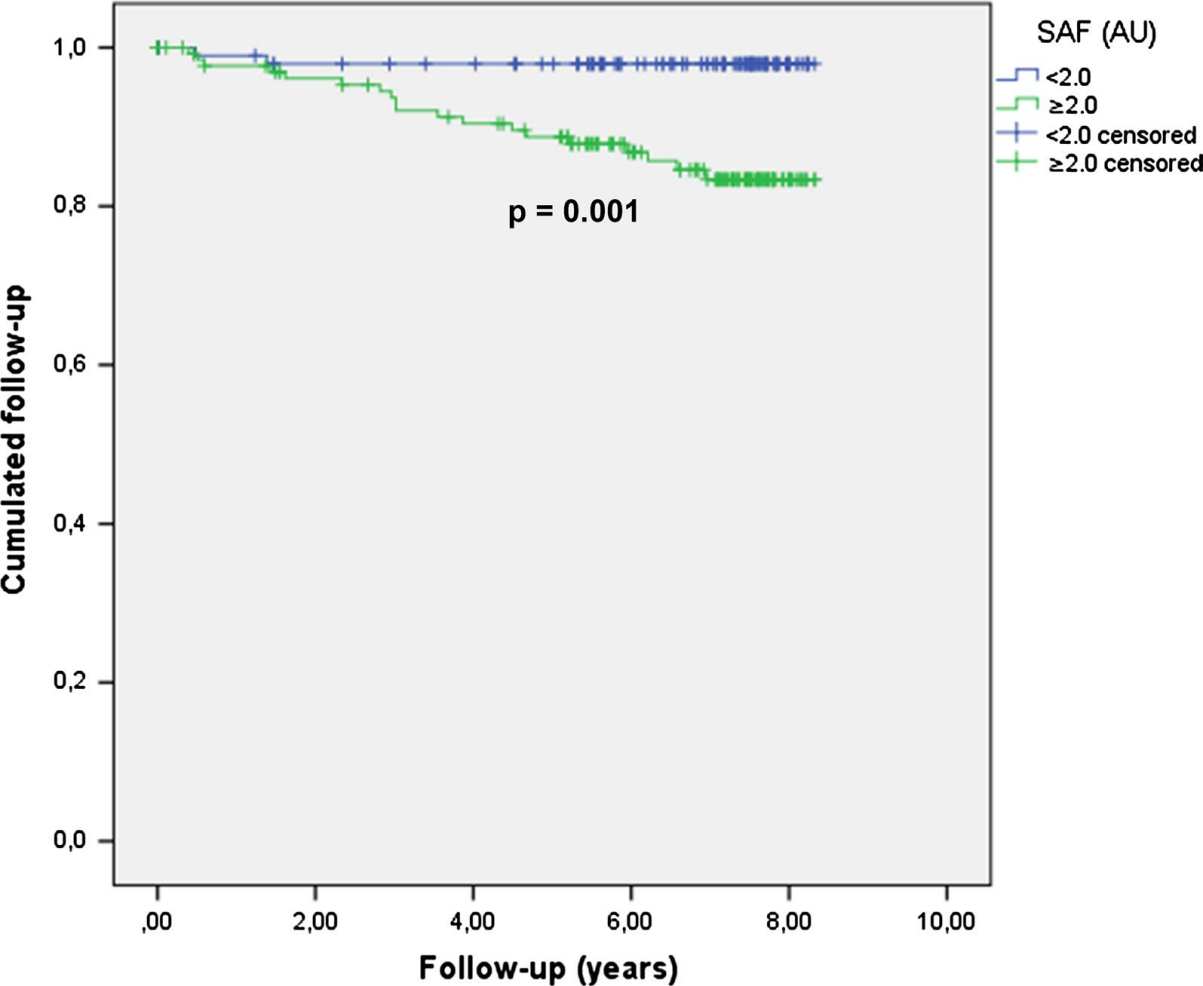
MACE during follow-up according to SAF median in patients with SAF < 2.0 and patients with SAF 2.0. p = 0.001

## Discussion

Higher SAF measured in 2009 was associated with increased risk of later cardiovascular events (HR: 4.13, CI 95%: 1.30–13.07) after adjusting for diabetes duration, arterial hypertension, treatment by statins, renal parameters and previous cardiovascular events. SAF therefore appears as a significant predictor of cardiovascular events in Type 1 Diabetes. Participants with DT1 who experienced a cardiovascular event during the 7 years follow-up were older, with a 9 years longer diabetes duration, but similar HbA1C as compared to the others. They had more arterial hypertension, more dyslipidemia according to similar blood lipids despite twice more treated by statins, more frequent abnormal albumin excretion and estimated glomerular filtration rates, and more previous cardiovascular events. Their SAF were + 30% higher.

### SAF to predict MACE

The prediction of cardiovascular events by SAF has already been reported in other diseases, as HIV infection [16] and Chronic Kidney Diseases although significance was lost (p = 0.09) after multivariate adjustments [17]. In type 2 diabetes, Lutgers et al. have shown that a high SAF (upper than median) was related to the 3 years cardiovascular prognosis, adjusted to the UKPDS risk score [13]. For Type 1 Diabetes, cross-sectional studies have reported relationship between SAF or skin intrinsic fluorescence and coronary calcifications [18], and clinical history of coronary heart disease [19]. But no prospective study had shown that SAF could predict cardiovascular events. In our previous analysis on the same cohort with a shorter follow-up of only 4 years [14], SAF predicted later eGFR impairement and later MACE: OR 4.84 (1.31–17.89), but the relation with later MACE did not reach significance with adjustment for previous MACE: OR 2.98 (0.77–11.47). However, this previous analysis was based on lower cases of cardio-vascular events. Three years later, the relation between SAF keeps significant after this adjustment: OR 4.13 (1.30–13.07). The longer follow-up with higher numbers of events now allows to reach significance for the prediction of macrovascular events in our patients independently from classical risk factors.

### The role of AGEs

The main mechanism underlying this prediction is the deleterious effect of AGEs on the vascular system [9]. Previous studies found that serum levels of advanced glycation end-products-modified LDL [20], and Methylglyoxal [21] were related to later cardiovascular events or to the progression of atherosclerosis as reflected by the carotid intima-media thickness, in type 1 diabetes. AGEs are generated inside peripheral cells before they are liberated in the extracellular space [9]. Their accumulation in tissues as estimated by SAF is expected to better reflect their influence, than their circulating levels. SAF has been related to cardiac tissue glycation [22], inappropriate left ventricular mass and diastolic dysfunction [23], long wavelenght-1 and sublinical markers as the intima-media thickness [24] and it is increased in patients with an abdominal aortic aneurysm [25]. The skin concentrations of AGEs have been related to coronary calcifications, left ventricular masses and progression of intima-media thickness in the DCCT-EDIC study [11]. SAF was related to the age of the participants (ß = + 0.397; p < 0.001) which is very similar to reports from other groups in participants with T1D [26–28]. Although an indirect estimation of the accumulation of AGEs, SAF has some advantages on skin biopsies: low cost, simplicity, non-invasiveness, and repeatability.

### SAF and metabolic memory

The SAF may predict macrovascular events because it is a marker of the metabolic memory of hyperglycaemia, which seems critical for macroangiopathy in T1D. The cardiovascular benefits of the intensive glucose control during the DCCT came apparent only later, 11 years after the end of its active phase [7], which led to propose the concept of glucose memory. This concept is also supported by the long-term results of the UKPDS for type 2 diabetes. A recent analysis of the risk factors for cardiovascular disease in the DCCT-EDIC participants has emphasized the major role of their mean time-weighted HbA1C, second only to age [8]. Previous studies have reported that SAF relates to the HbA1C of the previous years, better than to the present HbA1C in T1D [27, 29–31]. In elderly participants of the 3-City cohort study from the general population, we have shown that SAF relates to glycaemia and HbA1C measured ten years before, rather than to their values measured at the same time as SAF [32]. Most of the associations between skin intrinsic fluorescence and T1D complications lost significance after adjustment to the mean HbA1C over time for the DCCT participants [33]. The association between skin fluorescence and coronary arterial calcifications was however still significant after this adjustment, arguing for an additional value of the fluorescence measurement. But no prospective study had shown that SAF could predict cardiovascular events. In our previous analysis on the same cohort with a shorter follow-up of only 4 years [14], SAF predicted later eGFR impairement and later MACE: OR 4.84 (1.31–17.89), but the relation with later MACE did not reach significance with adjustment for previous MACE: OR 2.98 (0.77–11.47). Three years later, the relation between SAF keeps significant after this adjustment: OR 4.13 (1.30–13.07). In the real life, the history of HbA1C is often not complete for many T1D patients, and death from cardiovascular causes are thrice more frequent even for the best controlled (HbA1C ≤ 6.9%) according to the Swedish register [6]. This underlines the interest of a metabolic memory marker that predicts cardiac events.

### Others factors

Hyperglycaemia-related mechanisms may not be the only explanation for the relationship between SAF and macrovascular diseases. SAF increases with age, the strongest risk factor for cardiovascular events in T1D [8]. SAF has recently been related to the components of the metabolic syndrome [34], especially low HDL-cholesterol and arterial hypertension [35], that increasingly mediate the effect of glucose control on cardiovascular risk in aging T1D [5]. The progression of SAF in our patients with T1D highly depends on their glomerular filtration rates [15], as expected because renal insufficiency reduces the clearance of AGEs. This effect probably worsens as SAF predicts the impairment of GFR in T1D [14]. Diabetic nephropathy is a well-known, major contributor to cardiovascular disease in T1D [36]. But the relation between SAF and cardiovascular events reported in our study, was still significant after adjusting for age, arterial hypertension, blood lipids and renal parameters.

## Limitations

Some other hypothesis can however not be excluded on the basis of our study, which can be considered as limitations. We have recently shown that SAF quickly rises during acute renal failure [37]. That is a powerful predictor of major cardiovascular events in type 2 diabetes [38], but to our knowledge this has not been reported for T1D. We also found that SAF progressed less in our patients whose T1D was treated by continuous subcutaneous insulin infusion CSII [15], which has been related to less cardiovascular mortality [39]. This suggests that glycaemic variability may play a role in the accumulation of AGEs: even transient pikes of hyperglycaemia may have deleterious effects on vascular cells [10]. Long-term glycemic variability has been related to a twice-higher risk of cardiovascular events in T1D [40]. Skin autofluorescence is however not influenced by short-term glycemic variations [41]. Finally, some reports have related SAF to physical activity indexes: aerobic exercise capacity, muscle strength [42], and regular performance of physical activities [43, 44], which has been related to less cardiovascular events in the FinnDiane cohort [45]. Some practical limitations must also be mentioned. According to French law, we did not categorize the participants according to their ethnicity, but some subjects were excluded due to their skin pigmentation as already mentioned. The measurement of SAF were performed on intact skin, but we can not rule out that some subjects might have applied some body lotions locally. Draelos et al. applied a topical AGE inhibitor (blueberry extract) on the skin of 20 women with diabetes during three months, and they did not report any change of their SAF on this time interval [46]. An important proportion of the variability of SAF is not explained by age and usual covariates, some studies have suggested that genetic [47] or dietary factors may play a role, as diet is a source of AGEs [48]. Smoking has been related to SAF [35] and the relation between SAF and MACE was adjusted for tobacco use in our study, but we did not collect detailed dietary informations, so we can not exclude the role of some specific diet components, like coffee [35].

## Conclusion

SAF seems to be a good mean to predict MACE in T1D. Further works are now required to explore these hypothesis with more long term follow-up because fortunately only a few MACE occurred, a multicentric study with more patients is required to find strategies to slower the progression of SAF in T1D.

## Additional file

**Additional file 1.** Scatterplot for SAF over age.

## Abbreviations

AER: albumin excretion rate
AGE: advanced glycation end-products
AU: arbitrary unit
BMI: body mass index
CSII: continuous subcutaneous insulin infusion
DCCT: diabetes control and complications trial
eGFR: estimated glomerular filtration rates
HbA1c: glycated haemoglobin
HIV: human immunodeficiency virus
HR: hazard ratio
IQR: interquartile range
MACE: major adverse cardiovascular event
SAF: skin autofluorescence
SD: standard deviation
T1D: type 1 diabetes
UKPDS: United Kindom Prospective Diabetes Study
