# Supplementary material for: Skin autofluorescence predicts major adverse cardiovascular events in patients with type 1 diabetes: a 7-year follow-up study
Source: Cardiovasc Diabetol. 2018 Jun 8;17:82. doi: 10.1186/s12933-018-0718-8 (PMC5993997; doi:10.1186/s12933-018-0718-8)
Supplement: Supplementary file 1 [file 12933_2018_718_MOESM1_ESM.docx]

Age (year)

SAF

Prediction interval

95% Confidence lnterval

Regression

SAF
